# Spontaneous head twitches in aged rats: behavioral and molecular study

**DOI:** 10.1007/s00213-022-06253-y

**Published:** 2022-10-24

**Authors:** Alicja Zakrzewska-Sito, Przemysław Bieńkowski, Marcin Kołaczkowski, Irena Nalepa, Agnieszka Zelek-Molik, Adam Bielawski, Katarzyna Chorążka, Julita Kuczyńska, Paweł Mierzejewski

**Affiliations:** 1 Department of Pharmacology, Institute of Psychiatry and Neurology, ul. Sobieskiego 9, 02-957 Warsaw, Poland; 2 Department of Psychiatry, Warsaw Medical University, Nowowiejska 27, 00-665 Warsaw, Poland; 3 Department of Medicinal Chemistry, Faculty of Pharmacy, Jagiellonian University Medical College, Medyczna 9, 30-688 Kraków, Poland; 4 Department of Brain Biochemistry, Maj Institute of Pharmacology, Polish Academy of Sciences, Smętna 12, 31-343 Kraków, Poland

**Keywords:** Spontaneous head twitches, Aged rats, Serotonin 5-HT_2A_ receptor, Psychosis

## Abstract

**Rationale:**

We have discovered that rats at the age of 18 months begin to twitch their heads spontaneously (spontaneous head twitching, SHT). To date, no one has described this phenomenon.

**Objectives:**

The purpose of this study was to characterize SHT pharmacologically and to assess some possible mechanisms underlying SHT.

**Methods:**

Wistar male rats were used in the study. Animals at the age of 18 months were qualified as HSHT (SHT ≥ 7/10 min observations) or LSHT (SHT < 7/10 min observations). Quantitative real-time PCR with TaqMan low-density array (TLDA) approach was adopted to assess the mRNA expression of selected genes in rat’s hippocampus.

**Results:**

HSHT rats did not differ from LSHT rats in terms of survival time, general health and behavior, water intake, and spontaneous locomotor activity. 2,5-dimethoxy-4-iodoamphetamine (DOI) at a dose of 2.5 mg/kg increased the SHT in HSHT and LSHT rats, while ketanserin dose-dependently abolished the SHT in the HSHT rats. The SHT was reduced or abolished by olanzapine, clozapine, risperidone, and pimavanserin. All these drugs have strong 5-HT2A receptor–inhibiting properties. Haloperidol and amisulpride, as antipsychotic drugs with a mostly dopaminergic mechanism of action, did not influence SHT. Similarly, escitalopram did not affect SHT. An in-depth gene expression analysis did not reveal significant differences between the HSHT and the LSHT rats.

**Conclusions:**

SHT appears in some aging rats (about 50%) and is permanent over time and specific to individuals. The 5-HT2A receptor strongly controls SHT. HSHT animals can be a useful animal model for studying 5-HT2A receptor ligands.

**Supplementary Information:**

The online version contains supplementary material available at 10.1007/s00213-022-06253-y.

## Introduction

Research on old rats allows the evaluation of the aging processes and changes associated with aging, including the effect of specific drugs (Aura and Riekkinen 2000; Cass et al. 2005; Foley et al. 2004; Hernandez et al. 2006). Studies on old rats are difficult to carry out due to the long breeding period, which is expensive and labor-intensive because rats live 2–3 years on average (Sengupta 2013). Both physical and behavioral changes are observed in old rats. Old animals have reduced mobility, reduced limb tension and strength, and poorer coordination (Wallace et al. 1980). They are also more fearful (Blanchard et al. 1988; Boguszewski and Zagrodzka 2002; Frussa-Filho et al. 1992), show less interest in prizes (Blanton et al. 1998; Inui-Yamamoto et al. 2017; Malatynska et al. 2012; Spear and Varlinskaya 2010), and have reduced thirst (Thunhorst and Johnson 2003).

In our studies, when observing the behavior of aging animals, we noticed that some rats spontaneously shake their heads (spontaneous head twitching, SHT) at about 18 months of age (Bieńkowski et al. 2014). So far, this phenomenon has gone unnoticed and has hitherto not been described in the literature. It is similar to the phenomenon observed in rodents after the administration of psychedelic substances (Schreiber et al. 1994; Vetulani et al. 1980). Young rats do not present such spontaneous behavior.

We decided to carry out a detailed behavioral and pharmacological characterization of the spontaneous head-twitching phenomenon in old rats. We assessed the stability of the SHT over time and its repeatability. We divided the animals into those presenting a high number of spontaneous head twitching (HSHT), and a low number of spontaneous head twitching (LSHT). Next, we searched for any differences between these animals in terms of their behavior, appearance, health status, and survival rate. It was hypothesized that HSHT animals would present more symptoms associated with aging, live shorter, and have gene expression changes associated with serotonergic transmission.

The neurobiology of head twitching has been the subject of many studies and there is no doubt that the activation of the 5-HT_2A_ receptor plays an essential role therein (Glennon et al. 1984; Vollenweider et al. 1998). This behavior was first observed in mice after the intravenous administration of lysergic acid diethylamide (LSD) (Keller and Umbreit 1956). A similar effect was demonstrated after the administration of the precursor of serotonin-5-hydroxytryptophan, 2,5-dimethoxy-4-bromoamphetamine (DOB) or 2,5-dimethoxy-4-iodomamphetamine (DOI), and quipazine, a 5-HT_2A_ and 5-HT_3_ receptor agonist (Handley and Singh 1986; Vetulani et al. 1980). Mice with 5-HT_2A_ knockout do not present head twitching (Gonzalez-Maeso et al. 2007). Because head twitching appears to be directly related to the activation of the serotonin 5-HT_2A_ receptor, we studied the effects of the 5-HT_2A_ receptor agonist DOI on the number of head twitches both in HSHT and LSHT animals, and we also checked the effects of ketanserin, a 5-HT_2A_ receptor antagonist, on the number of SHT in HSHT animals.

Antipsychotics with a strong affinity to the 5-HT_2A_ receptor risperidone or olanzapine, reduce DOI-induced head twitching (Amada et al. 2019; Dong et al. 2009). Hence, we decided to test the effects of the chosen antipsychotics: haloperidol (first-generation antipsychotic, FGA), amisulpride (second-generation antipsychotic, SGA; selective dopaminolytic), olanzapine (non-selective SGA), clozapine (atypical antipsychotic), risperidone (SGA, potent 5-HT_2A_ antagonist), pimavanserin (reverse 5-HT_2A_ agonist), escitalopram (selective serotonin reuptake inhibitor, SSRI; antidepressant) on SHT in the HSHT rats. It was hypothesized that 5-HT_2A_ antagonists would reduce the number of SHT.

In order to better understand the neurobiological basis of SHT, we decided to study the mRNA expression of genes encoding monoamine receptors, alpha and beta subunits of G proteins, small G proteins, adenyl cyclase, protein kinases, phospholipases, matrix metalloproteinases, and sirtuins in the hippocampus of old rats (Bielawski et al. 2018). The hippocampus was selected because it is an important structure involved in a number of processes related to aging, dementia, schizophrenia, and memory (Allen et al. 2007; Bettio et al. 2017; Opitz 2014; Tamminga et al. 2010). Moreover, the hippocampus is intimately connected with the medial prefrontal cortex (mPFC) through bidirectional monosynaptic and polysynaptic pathways (Taylor et al. 2016). And it was the mPFC that was proposed as one of the brain regions engaged in the head-shaking reaction (Willins and Meltzer 1997). So, we started our first screening of mRNA analysis in the hippocampus and we expected there to be differences in the expression of genes related to the serotonergic system, especially associated with the 5-HT_2A_ serotonin receptor.

## Materials and methods

### Animals

Male Wistar rats (Charles River, Sulzfeld, Germany) aged eighteen months or older were used for the study. Rats were housed in transparent, polycarbonate cages (3–4 animals per an area of 1760 cm^3^ depending on body weight) in rooms with standard, constant conditions of temperature (22 ± 2 °C), relative humidity (50%), and a 12:12 daily light/dark cycle (with lights on at 07:00 am). Access to tap water and food was unlimited (Labofeed B, WPIK, Kcynia, Poland). The cages were supplemented with wooden blocks. All rodents were weighed, handled, and monitored for health throughout their growth and aging period. During experiments the observer was blinded, another person administered the drug and another person made observations. The study was performed in full accordance with the ethical standards laid down in respective Polish and European regulations (Directive No. 2010/63/EU). The Local Committee for Animal Care approved all the experimental procedures (No. 27/2013 and No. 9/2014).

### Spontaneous head twitching

The rats were placed in glass observation cages (25 × 25 × 40 cm, W × H × L) with wood chip bedding on the floor. SHT was counted for 10 min (600 s) by a blinded observer. SHT was scored as described by Millan et al. (2000), with some minor modifications.

The stability of the SHT feature was assessed based on repeated observations over time. SHT observations were repeated after 24 h and 2 months. The studies were conducted on relatively numerous groups of animals (*n* = 32 and *n* = 29). All the observations were carried out in identical conditions, that is, in the same room and with the same lighting.

### Animal classification: division into LSHT/HSHT group

The animals were selected and divided into the LSHT and the HSHT group when they reached 18 months of age. The rats were screened for the presence of SHT in a 10-min pre-test. Rats showing ≥ 7 SHT/10 min (median split) were classified as HSHT, and the remaining animals were classified as LSHT.

### Observation of animals, comparison of LSHT and HSHT groups

LSHT (*n* = 16) and HSHT (*n* = 16) rats were monitored for body weight, food intake, water intake, spontaneous locomotor activity, and survival over a period of 18–22 months of age. Every two months, the rats were placed individually in cages with free access to food and water. After 3 days of acclimatization, the individual consumption of food and water was monitored over the next three days. Locomotor activity was assessed every two months in an open-field test.

An additional parameter that was assessed was the survival time of the HSHT and the LSHT rats. Close observations of the health status and changes in behavior were carried out on 12 HSHT rats and 8 LSHT rats from a total of 20 animals who lived to the age of 26 months. For this purpose, the animals were placed individually in plexiglass cages (41 × 25 × 25 cm) that were lined with bedding for 15 min. All the changes like an abnormal body posture, the presence of diarrhea, the occurrence of edema, movement disorders (impaired coordination, limping, walking backwards, rotation around own axis, and abnormal limb tension), the occurrence of tumors/lipomas, respiratory system disorders, and aggression were noted.

### Open-field test

Locomotor activity was assessed in black octagonal cages (80 cm in diameter, 30 cm high). Each animal was placed in the central part of the open field and allowed to freely explore the whole area for 30 min. Forward locomotion (cm/30 min) was registered and analyzed with the aid of a video tracking system (Videomot, TSE Systems, Germany).

### Assessment of DOI impact on head twitching in LSHT and HSHT rats and ketanserin on head twitching in HSHT rats

DOI (2,5-dimethoxy-4-iodoamphetamine) (Cayman, USA), a potent 5-HT_2A_ serotonin receptor agonist, was dissolved in physiological saline (0.9% NaCl, Baxter, Poland) and administered intraperitoneally (i.p.) to the HSHT and LSHT rats (*n* = 8/group) at a dose of 2.5 mg/kg in a volume of 1.0 ml/kg 5 min before the test (Kołaczkowski et al. 2014a). Ketanserin (Cayman), a 5-HT_2A_ serotonin receptor antagonist, was dissolved in physiological saline and administered i.p. to only HSHT rats (*n* = 8/group) at doses of 0.1, 0.3, and 1.0 mg/kg in a volume of 2.0 ml/kg 30 min before the experiment (Cui et al. 2018).

### Estimation of psychotropic drugs’ influence on SHT in the group of HSHT rats

The following drugs were used to pharmacologically evaluate the HSHT model of psychosis: haloperidol (selective first-generation antipsychotic), amisulpride (selective second-generation antipsychotic), olanzapine (non-selective second-generation antipsychotic), clozapine (atypical antipsychotic), risperidone (second-generation antipsychotic, potent 5-HT_2A_ receptor antagonist), pimavanserin (5-HT_2A_ inverse agonist approved for the treatment of Parkinson’s disease psychosis), and escitalopram (selective serotonin reuptake inhibitor, potent antidepressant). Haloperidol (Polfa Warszawa S.A., Poland) was dissolved in 0.9% NaCl and administered s.c. at doses of 0.01, 0.03, and 0.1 mg/kg in a volume of 1.0 ml/kg 60 min before the test; amisulpride (Cayman) was suspended in a 1.5% aqueous solution of Tween 80 (Sigma-Aldrich) and administered i.p. at doses of 1.0, 3.0, 10.0, and 30.0 mg/kg in a volume of 2.0 ml/kg 60 min before testing; olanzapine (Cayman) was suspended in a 1.5% aqueous solution of Tween 80 (Sigma-Aldrich) and administered i.p. at doses of 0.1, 0.3, and 1.0 mg/kg in a volume of 2.0 ml/kg 60 min before testing; clozapine (Sigma-Aldrich) was dissolved in a 1.5% aqueous solution of Tween 80 with a few drops of acetic acid and administered i.p. at doses of 0.3, 1.0, and 3.0 mg/kg in a volume of 3 ml/kg 60 min before the test; risperidone (Cayman) was suspended in a 1.5% aqueous solution of Tween 80 and administered i.p. at doses of 0.1, 0.3, and 1.0 mg/kg in a volume of 1.0 ml/kg 60 min before testing; pimavanserin (Cayman) was dissolved in 0.9% NaCl and administrated i.p. at doses of 1.0, 3.0, and 10.0 mg/kg in a volume of 2.0 ml/kg 60 min before the experiment; escitalopram (Sigma-Aldrich) was dissolved in physiological saline and administrated i.p. at doses of 1.0, 3.0, and 10 mg/kg in a volume of 2.0 ml/kg 60 min before the test. All the solutions were prepared immediately prior to use. The animals were placed individually in 41 × 25 × 25 cm plexiglass cages and the number of spontaneous head twitches over a 10 min period was counted.

### mRNA analysis of gene expression

To search for the mechanisms of spontaneous neurodegeneration in the hippocampus of old rats, we compared the expression of 94 genes listed in Suppl. Table 1 (Tab. S1), in the hippocampus of old rats (LSHT and HSHT groups; 26 months old) to the level of these genes in adult rats (3 months old). After performing behavioral tests, old rats without pharmacological treatments: LSHT (*n* = 5) and HSHT (*n* = 8), as well as the control group (composed of 3-month adult rats, *n* = 9) rats were anesthetized with pentobarbital (80.0 mg/kg) and decapitated. The isolated hippocampi were immediately placed in RNAlater, a ribonucleic acid (RNA) protecting and stabilizing solution, and stored in a freezer at a temperature of − 20 °C until analyzed. The total RNA was isolated and purified based on a previously described protocol (Zelek-Molik et al. 2019). The tissue was placed in a lysis buffer containing guanidinium thiocyanate (Qiagene, USA) in a volume of 0.4 ml/20 mg of tissue, and homogenized using high-speed shaking (30/s) in plastic tubes with stainless steel beads in TissueLyser II apparatus (Qiagene). The total RNA was purified using the RNeasy Mini kit (Qiagene). The quantity of RNA was determined spectrophotometrically at 260 nm and 260/280 nm (ND/1000 UV/Vis; Thermo Fisher NanoDrop, USA). The quality of RNA was determined using the RNA 6000 Nano LabChip kit and the Agilent Bioanalyzer 2100 (Agilent, USA). Only good quality samples, with RNA integrity number (RIN) > 7.5 values, were chosen for mRNA expression analysis. The RT reaction was performed at a final volume of 20 μl with 300 ng of RNA (as a cDNA template) using the High-Capacity cDNA Reverse Transcription kit (Applied Biosystems, USA) according to the manufacturer’s protocol. The products of the RT reaction were amplified using the TaqMan Gene Expression Master Mix (Applied Biosystems, USA). Reverse transcription and the quantitative polymerase chain reaction (RT-qPCR) were performed using the QuantStudio™12 K Flex system (Life Technologies, USA).

The expression of mRNA for 94 different genes, was measured first using custom TaqMan array cards and the low-density array (TLDA) method (*n* = 8 HSHT, *n* = 5 LSHT, *n* = 9 control), according to the manufacturer’s protocol on the QuantStudio™ 12 K Flex system (Life Technologies, USA). The relative quantification (ΔΔCt) method was used for the analysis of the TLDA data. The obtained TLDA results were verified by further detailed analyses on three groups of rats (*n* = 8 HSHT, *n* = 5 LSHT, and *n* = 9 control) using PCR with single TaqMan probes for the most interesting candidates chosen based on TLDA analysis. HPRT and β2m served as the reference genes. In the case of the single assays, the products of the RT reaction were amplified using the TaqMan Gene Expression Master Mix (Applied Biosystems, USA) in a total volume of 10 µl containing the 1 TaqMan Gene Expression Master Mix, 30 ng of cDNA (used as the PCR template), and 250 nM of the TaqMan probe labeled with FAM as the fluorescent dye. The standard curve method was used for the single TaqMan probe assays.

### Data analysis

The statistical analysis was carried out using the STATISTICA v.13.1 software (Statsoft), and the GenE × 5.7 software was additionally used for the analysis of gene expression.

Two-way ANOVA with repeated measures (group × time) test was used for the comparison of the HSHT and LSHT rats in terms of the number of spontaneous head twitches, body weight, food consumption, the amount of water intake, and locomotor activity over time. The effect of DOI was analyzed using two-way ANOVA (group × dose). One-way ANOVA was used to analyze the effect of ketanserin, haloperidol, amisulpride, olanzapine, clozapine, risperidone, and pimavanserin on spontaneous head twitching in the HSHT rats. The Newman-Keuls test was used for post hoc comparisons.

The Cox-Mantel test was used to evaluate the differences in the survival of the HSHT and the LSHT rats. Pearson’s correlation coefficients were calculated to evaluate the repeatability of the SHT.

TLDA data analysis: the data were exported from the QuantStudio12K Flex Software v1.4 for detailed analysis using the GenEx Pro 5.7 software (MultiD Analyses AB, Sweden). Since the geNorm and the NormFinder algorithms indicate that the endogenous control genes (18S and B2m) applied in this study are unsuitable, the global normalization method was used. One-way ANOVA followed by the Tukey–Kramer test was applied to select the genes that were particularly interesting for further verification using the single TaqMan probe assays.

Single TaqMan probe data analysis: one-way ANOVA and HSD for unequal *N* (a modification of the Tukey honesty significant difference (HSD) test) were used to compare the gene expression between the HSHT group, the LSHT group, and the group of young rats (control group). Student’s *t*-test was used to compare old (pooled HSHT and LSHT groups) and young rats.

*P* values of < 0.05 were considered significant.

The STATISTICA v.13.1 software (Statsoft) and Microsoft Excel were used to create the artwork.

## Results

**Fig. 1:**
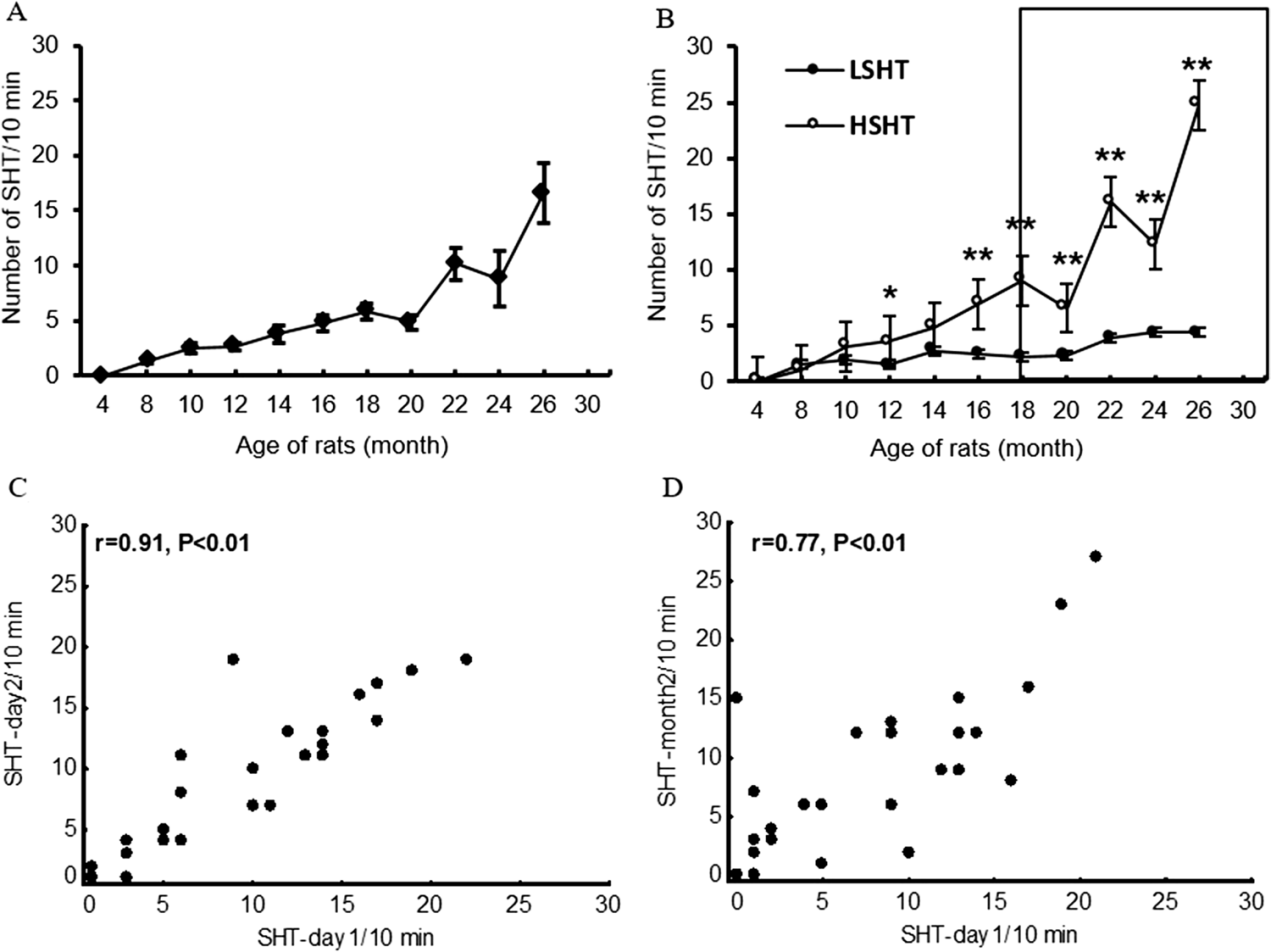
Spontaneous head twitching (SHT) in aging rats and their correlation. Spontaneous head twitching (SHT) in aging rats (**A**), at 18 months of age, the animals were divided into low spontaneous head-twitching rats (LSHT, SHT < 7/10 min), and high spontaneous head-twitching rats (HSHT, SHT ≥ 7/10 min) (**B**). The results are presented as the mean ± SEM; *n* = 32. *P < 0.05, **P < 0.01 (HSHT vs LSHT). Correlation of SHT in aged rats (≥ 18 months of age)—day 1 vs day 2 (**C**), day 1 vs. day 60 (**D**)

The study revealed that there is an increase in spontaneous head twitching in aging rats (Fig. 1A) and that rats can be classified as demonstrating low spontaneous head twitching and high spontaneous head twitching based on SHT observance at 18 months of age (Fig. 1B). Experiments performed, confirmed that such a criterion works i.e. HSHT rats show a stable number of HT over time. All the results presented here are highly reproducible. The time of 18 months was chosen arbitrarily, based on observations of behavioral changes over time. Furthermore, our study confirmed that the chosen time point allows to determine which animals are LSHT and HSHT. Two-way ANOVA (group × time) revealed that there was a significant difference between the LSHT and the HSHT in terms of the SHT: F(1, 18) = 13.61, *P* < 0.05; a significant time effect F(9, 162) = 16.55, *P* < 0.01, as well as a significant group × time interaction F(9, 162) = 8.52, *P* < 0.01.

The SHT was stable over time. There was a strong correlation between the SHT measured on day 0 and day 1 (*r* = 0.91), and on day 0 and day 60 (*r* = 0.77) (Fig. 1C and D).

**Fig. 2:**
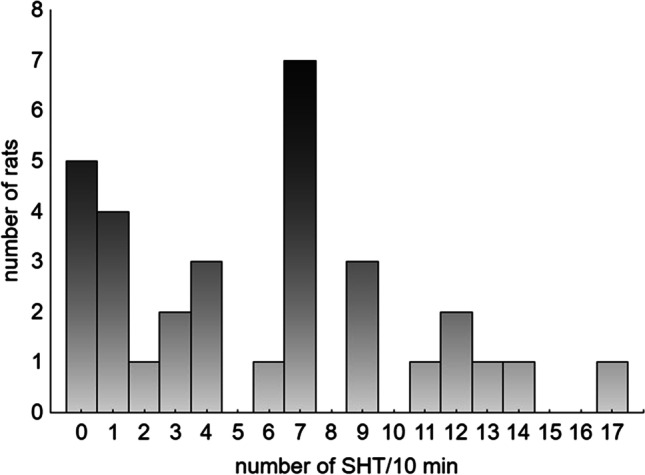
Distribution of spontaneous head twitches in 18-month-old animals (*n* = 32). Median = 6.5, rats with SHT ≥ 7 were classified as HSHT, rats with SHT < 7 were classified as LSHT

The distribution of SHT in 18-month-old animals indicates that eligibility criterion ≥ 7 is reasonable (Fig. 2). The median SHT was 6.5, the distribution was irregular and two-tailed, and the dominant value was 7, followed by the most common values of 0 and 1.

The LSHT and the HSHT rats did not differ in the survival time (Cox-Mantel test, *P* > 0.05), the body weights over time (2-way ANOVA, group × time F(6, 150) = 1.44, *P* > 0.05), the general health and behavior parameters (chi-square, all *P* > 0.05), the food intake over time (2-way ANOVA, group × time F(6, 150) = 0.27, *P* > 0.05), and the water intake (2-way ANOVA, group × time F(6, 150) = 1.13, *P* > 0.05, as well as spontaneous locomotor activity (2-way ANOVA, group × time F(6, 150) = 0.70, *P* > 0.05).

**Fig. 3:**
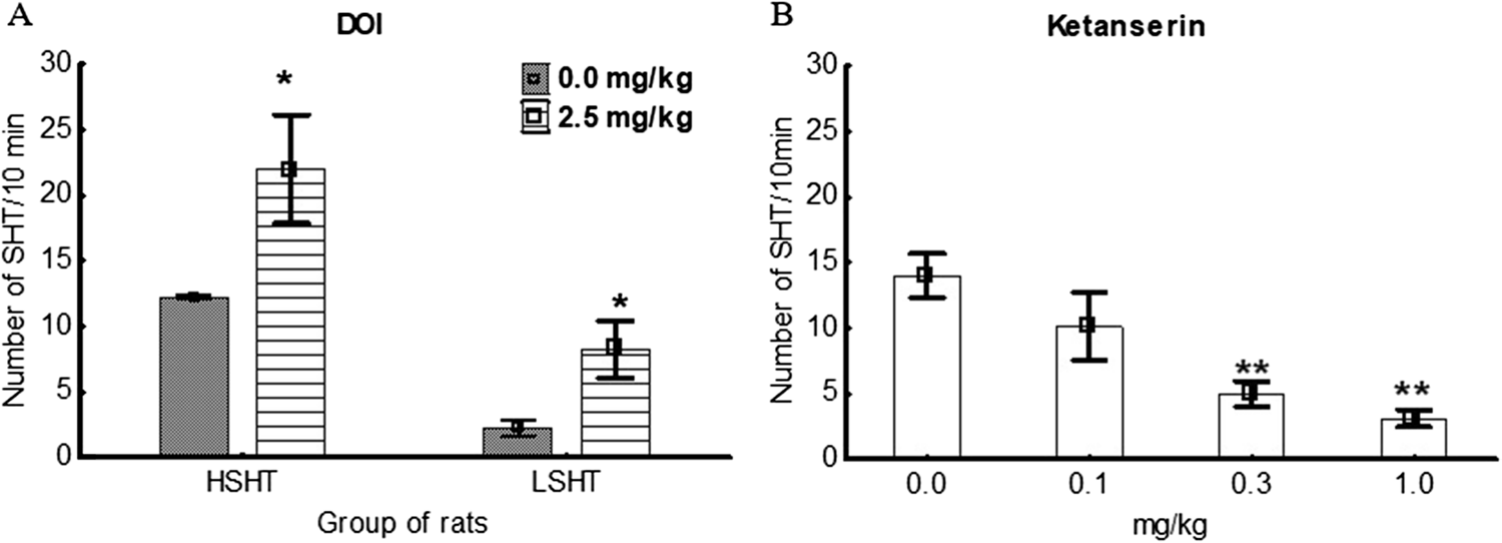
Effect of DOI (2,5-dimethoxy-4-iodoamphetamine) 2.5 mg/kg i.p. on head twitching in HSHT (high spontaneous head twitching) and LSHT (low spontaneous head twitching) rats (**A**). Effects of ketanserin, dose–response study, on head twitching in HSHT rats (**B**). The results are presented as the mean ± SEM. * *P* < 0.05, ** *P* < 0.01, Newman-Keuls post hoc test

DOI, a potent serotonin 5-HT2A receptor agonist, at a dose of 2.5 mg/kg, significantly increased the number of head twitches both in the HSHT and the LSHT rats (Fig. 3A). A 2-way ANOVA (group × DOI) revealed significant group effect F(1,28) = 22.7, *P* < 0.01, DOI effect F(1,28) = 10.0, *P* < 0.01, and insignificant interaction Group × DOI F(1,28) = 0,6, *P* < 0.05. Ketanserin, a non-selective serotonin 5-HT2 receptor antagonist, dose-dependently decreased the number of head twitches in the HSHT rats F(3, 28) = 8.10, *P* < 0.01 (Fig. 3B).

**Fig. 4:**
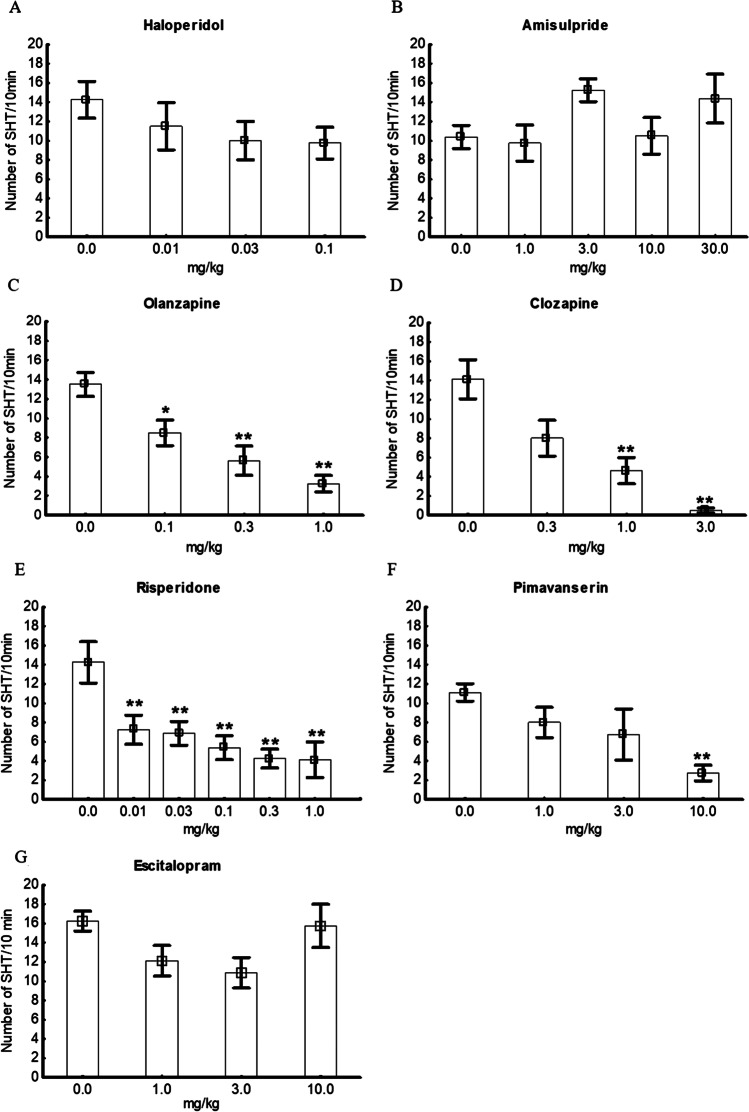
Effect of haloperidol, F(3, 28) = 0.94; *P* > 0.05 (**A**), amisulpride, F(4, 35) = 1.79; *P* > 0.05 (**B**), olanzapine, F(3, 28) = 11.20; *P* < 0.01 (**C**), clozapine, F(3, 28) = 12.49, *P* < 0.01 (**D**), risperidone, F(5, 41) = 5.39; *P* < 0.01 (**E**), pimavanserin, F(3, 28) = 3.92; *P* < 0.05 (**F**), and escitalopram, F(3, 28) = 2.28, *P* > 0.05 (**G**) on the number of SHT (spontaneous head Twitches) in the group of HSHT (high spontaneous head twitching) rats. The results are presented as the mean ± SEM. * *P* < 0.05, ** *P* < 0.01, Newman-Keuls post hoc test

The effects of chosen drugs on the SHT were tested (Fig. 4). Haloperidol (a D_2_, D_3_ antagonist), at doses of 0.01–0.1 mg/kg i.p., did not influence the number of the SHT in the HSHT rats, F(3, 28) = 0.94, *P* > 0.05. Similarly amisulpride (a D_2_, D_3_ antagonist), at doses of 1.0–30.0 mg/kg i.p., had no effect F(4, 35) = 1.79, *P* > 0.05. Olanzapine (a 5-HT_2A_, H_1_, D_2_, D_3_, M_1_, M_3_, H_1_ antagonist), at doses of 0.1–1.0 mg/kg i.p., dose-dependently decreased the number of the SHT, F(3, 28) = 11.20, *P* < 0.01. Clozapine (a H_1_, M_1_, 5-HT_2A_, 5-HT_2C_, 5-HT_7_, D_2_, D_3_ antagonist), at doses of 0.3–3.0 mg/kg, dose-dependently reduced the number of the SHT, F(3, 28) = 12.49, *P* < 0.01. Risperidone (a 5-HT_2A_, D_2_, D_3_, 5-HT_7_ antagonist), at doses of 0.01–1.0 mg/kg i.p., dose-dependently reduced the number of the SHT, F(5, 41) = 5.39, *P* < 0.01. Pimavanserin (a 5-HT_2A_ inverse agonist), at doses of 1.0–10.0 mg/kg i.p., significantly decreased the SHT, F(3, 28) = 3.92, *P* < 0.05; a significant reduction in the SHT was observed at a dose of 10.0 mg/kg i.p. Escitalopram (an SSRI) did not influence the SHT, F(3, 28) = 2.28, *P* > 0.05.

### Analysis of the mRNA expression of selected genes

A statistically significant difference in the expression of the studied genes between the investigated groups of animals occurred in the case of 14 out of the 94 analyzed genes (Supplementary Fig. 1A – Fig. S1A). The genes coding for β_1_-adrenergic receptor (ADRB1), β_2_-adrenergic receptor (ADRB2), 5-hydroxytryptamine 1A receptor (HTR1A), 5-hydroxytryptamine 5B receptor (HTR5B), metallopeptidase 2 (MMP2), mitogen-activated protein kinase 8 (MAPK8), phospholipase C type β_2_ (PLCB2), and β_C_ protein kinase (PRKCB) were selected for further verification using single TaqMan probes. The one-way ANOVA results of the mRNA expression of all the 94 analyzed genes in the rats’ hippocampi are summarized in Suppl. Table 2 (Tab. S2).

In the single TaqMan probe assay validation, the genes encoding hypoxanthine phosphoribosyltransferase (HPRT) and β_2_-microglobulin (β2M) were used as reporter genes. The expression of the reporter genes underwent statistically significant changes; therefore, the results were presented without prior standardization to the reporter gene (one-way ANOVA for HPRT: F(2, 19) = 5.64, *P* < 0.05; the HSD test for unequal *N*: *P* > 0.05 for HSHT vs control; *P* < 0.05 for LSHT vs control; *P* > 0.05 for HSHT vs LSHT, and one-way ANOVA for β2M F(2, 19) = 10.36, *P* < 0.01, the HSD test for unequal *N*: *P* < 0.01 for HSHT vs control; *P* > 0.05 for LSHT vs control; and *P* > 0.05 for HSHT vs LSHT).

Ultimately, in-depth gene expression analysis revealed no statistically significant differences between the HSHT and the LSHT rats, but differences were noted between the aged (HSHT and LSHT) and the young (control) animals for the following genes: ADRB2 (*t* =  − 3.28; df = 19; *P* < 0.01; *n* = 21), HTR1A (*t* =  − 3.60; df = 20; *P* < 0.01; *n* = 22), HTR5B (*t* = 5.03; df = 20; *P* < 0.01; *n* = 22), MMP2 (*t* = 7.84; df = 20; *P* < 0.01; *n* = 22), MAPK8 (*t* = 5.90; df = 20; *P* < 0.01; *n* = 22), and PRKCB (*t* =  − 6.30; df = 20; *P* < 0.01; *n* = 22) (Suppl. Fig. S1B).

## Discussion

The phenomenon of SHT increases with age, and in 18-month-old rats, it is possible to clearly identify those that show a high number of SHT (SHT ≥ 7/10 min) and those that demonstrate a small number of SHT (SHT < 7/10 min). Our studies revealed that this trait is stable over time and is individually specific. The division into HSHT and LSHT groups was carried out at 18 months of age, which is a time when rats can be considered old and when significant individual differences in the number of SHT are observed. Additional studies undertaken by us showed that the HSHT and LSHT groups do not differ significantly in body weight, amount of food and water consumed, health status, survival, behavior, and locomotor activity. According to our observations, the survival rate of old animals, both in the case of the LSHT and the HSHT, do not differ significantly from literature data (Turturro et al. 1999; Yorke et al. 2017).

In our opinion, DOI-induced head twitches are not a stereotypic-like behavior but it is more like nervous tics. There are studies showing that DOI injections (2.5 mg/kg) increased dopamine release in the rat cortex (Huang et al. 2010); however, we did not observe any dopamine-related stereotypic-like behaviors in rats after DOI administration (sniffing activity, gnawing behaviors, locomotor stereotypy, etc.). There are very few studies describing the effects of DOI in old and young animals. In mice, with regards to age, the dose–effect curve for DOI was shifted slightly downward (i.e., DOI was less effective) in older (3–3½-month-old) animals relative to younger (5–6-week-old) animals (Weiss et al. 2003). In fact, our study is the first one describing the effects of DOI on 18-month-old rats.

Spontaneous HT can phenotypically resemble tics or other movement disorders like Parkinson’s disease, that worsen with age. On the other hand, however, head twitching identical to SHT occurs after the administration of psychedelic substances such as DOI or LSD. For this reason, DOI is used in pharmacological studies to model the positive symptoms of schizophrenia, and head twitches are then considered equivalent to positive symptoms like hallucinations. (Rogóż 2012). The DOI-elicited HT has been considered an animal model of a variety of behavioral and psychiatric conditions including hallucinogenesis, schizophrenia, obsessive–compulsive disorders, Tourette’s syndrome, and more generally as a model of 5-HT2 receptor function/activity (Canal and Morgan 2012). SHTs appearing with age are clearly associated with 5HT_2A_ receptor activity; hence, per analogiam in our opinion, this phenomenon models psychosis disorders rather than movement disorders. DOI is a psychogenic, hallucinogenic substance, and the increase in the number of HT after DOI can be considered a DOI-induced psychosis (Halberstadt and Geyer 2013). It is possible that SHT and DOI-induced HT are related to a common mechanism involving the overstimulation of the 5HT_2A_ serotonin receptor (Kołaczkowski et al. 2014b; McFadden et al. 2018; Schreiber et al. 1995). This assumption was confirmed by the administration of ketanserin, a selective 5-HT_2A_ antagonist, which dose-dependently significantly reduced SHT in the HSHT animals. It can therefore be assumed that the development of SHT in aging animals is associated with changes in the activity of the serotonergic system.

However, this hypothesis was not confirmed by analysis of mRNAs expression of genes encoding for monoaminergic receptors and their downstream effectors, which did not show significant differences in the expression of genes associated with the serotonergic system between the HSHT and the LSHT rats. It is important to note that we only examined one brain structure, the hippocampus. This structure was chosen because the hippocampus is part of the limbic system, which is primarily responsible for memory and may be related to psychosis. Moreover, a dysfunction of the hippocampus is observed not only in Alzheimer’s disease but also in schizophrenia. Apart from that this brain region and the mPFC possess reciprocal connections through bidirectional monosynaptic and polysynaptic pathways (Taylor et al. 2016) and the mPFC was suggested to play role in the head-shaking reaction (Willins and Meltzer 1997). It is possible that the age-related changes in serotonergic transmission may only occur in certain brain regions. However, to date, the region of the brain responsible for the head-shaking reaction has not been identified. Only single studies indicate that this may be the prefrontal cortex or the pars compacta of the substantia nigra (McFarland et al. 2011; Willins and Meltzer 1997). The direct administration of the serotonergic 5HT_2A_ agonist DOI or partial agonist (m-chloro-phenylpiperazine) into the medial prefrontal cortex of rats produced head twitching. The administration of the 5-HT_1A_ receptor agonist 8-OHDPAT blocked DOI-induced HT, probably due to the functional interaction between the 5-HT_1A_ and the 5-HT_2A_ receptors. It has also been reported that the bilateral lesion of substantia nigra induces head twitching and this can be blocked by the administration of a selective 5-HT_2A_ inverse agonist, pimavanserin (McFarland et al. 2011).

In our study, we also assessed the effects of selected drugs such as haloperidol, amisulpride, olanzapine, clozapine, risperidone, pimavanserin, and escitalopram on the number of head twitches in HSHT animals. We were interested in investigating whether drugs with confirmed antipsychotic efficacy would affect the phenomenon of SHT in old animals. A significant decrease in SHT was observed after the administration of olanzapine at doses of 0.1, 0.3, and 1.0 mg/kg, clozapine at doses of 1.0, and 3.0 mg/kg, risperidone at doses of 0.01, 0.03, 0.1, 0.3, and 1.0 mg/kg, and pimavanserin at a dose of 10.0 mg/kg. Haloperidol, amisulpride, and escitalopram did not produce any significant effect on SHT. These results support the serotonergic hypothesis of SHT.

The effects of the drugs studied were rather specific and did not simply depend on their sedative properties, e.g., haloperidol at a dose of 0.1 mg/kg had in our laboratory strong sedative effect but did not significantly affect SHT; on the other hand, clozapine at a dose of 1 mg/kg and risperidone in doses 0.01–0.1 did not affect locomotion and strongly inhibited SHT (see Kołaczkowski et al. 2014b).

To expand our understanding of the potential mechanisms underlying SHT, we studied the expression of 94 selected genes in the hippocampus. Young rats at 3 months of age were used as an additional control. When we were designing the TaqMan array card content and selecting genes to analyze their mRNAs expression, firstly we considered those related to serotonin receptors and their intracellular signaling pathways (e.g., phospholipase C and protein kinase C for 5HT2A receptor as well adenylate cyclase and PKA for other subtypes of 5HT receptors). However, it is well known that many functional interactions between serotonin and other transmitter systems, such as noradrenaline, dopamine, GABA, and glutamate exist (Adell et al. 2010). This made the analyses of receptors for other monoamines and their intracellular signaling components justified.

The preliminary analysis of obtained results revealed a significant difference between the LSHT and the HSHT rats and the control young animals in the expression of 14 genes coding the β_2_ adrenergic receptor, mitogen-activated protein kinase 8, protein kinase C β, phospholipase C β2, and RND2, one of the G-proteins in the GTPase family. Single TaqMan probes were used to further verify the selected genes and no differences were found between the LSHT and the HSHT rats. However, significant differences were shown between old rats (LSHT, HSHT) and young rats in the expression of genes coding for serotonergic receptors (HTR1A, HTR5B), metallopeptidase 2, protein kinases (MAPK8 and PRKCB). It was not the purpose of this study to compare gene expression between old and young animals. The decrease or increase in the gene expression of *ADRB2*, *HTR1A*, *HTR5B*, *MMP2*, *MAPK8*, and *PRKCB* in the group of old rats is probably age-related. Our results suggest that the adrenergic and serotonergic signaling pathways may significantly change with aging. Indeed the results are preliminary but to the best of our knowledge, such an analysis of the gene mRNAs expression of monoaminergic receptors and their related components of intracellular signaling in old rats have been shown for the first time by this study. Interestingly, the age-related biochemical changes demonstrated so far in rats include altered levels of neurotransmitters such as noradrenaline, dopamine, and serotonin in various brain regions including the hippocampus (Portero-Tresserra et al. 2020). Thus, it is very likely that the changes we observe in the gene mRNAs expression are somehow related to changing levels of monoamines. Our results have some limitations. First, the criterion for dividing into HSHT and LSHT rats was arbitrarily chosen by using a median split. Despite the clear effect of 5-HT_2A_ receptor, a neurobiological correlate could not be demonstrated. Analysis of genes’ mRNA expression showed no differences between HSHT and LSHT rats. Further studies are needed to determine whether the SHT phenomenon is actually related to changes in gene expression in other regions of the brain or results rather from disturbances in communication within neuronal networks, which may not necessarily be mirrored by specific changes in gene expression. We also do not know whether the observed SHT phenomenon in old rats is more common and applies to all rats or it is strain-specific and limited only to Wistar rats. It will be interesting to find out if it applies to other animals as well.

## Conclusion

In conclusion, the results of our observations confirm that SHT is a specific feature of some aging rats. This trait is clearly visible around 18 months of age. SHT are stable over time. They are strongly controlled by the 5HT_2A_ receptor as drugs that have an antagonistic effect on the 5HT_2A_ receptor cause a significant reduction in the number of SHT. HSHT animals can be a useful animal model for the study of 5HT_2A_ receptor ligands.

## Supplementary Information

Below is the link to the electronic supplementary material.

Supplementary file1 (PDF 817 KB)
