# Supplementary material for: Spontaneous head twitches in aged rats: behavioral and molecular study
Source: Psychopharmacology (Berl). 2022 Oct 24;239(12):3847–57. doi: 10.1007/s00213-022-06253-y (PMC9672005; doi:10.1007/s00213-022-06253-y)
Supplement: Supplementary file 1 — Supplementary file1 (PDF 817 KB) [file 213_2022_6253_MOESM1_ESM.pdf]

Supplementary materials

Spontaneous head twitches in aged rats: behavioral and molecular study

Alicja Zakrzewska-Sito[1], Przemysław Bieńkowski[2], Marcin Kołaczkowski[3], Irena Nalepa[4], Agnieszka Zelek-Molik[4], Adam Bielawski[4], Katarzyna Chorązka[4], Julita Kuczyńska[1], Paweł Mierzejewski[1]

[1] Department of Pharmacology, Institute of Psychiatry and Neurology, Sobieskiego 9, 02-957 Warszawa, Poland
[2] Department of Psychiatry, Warsaw Medical University, Nowowiejska 27, 00-665 Warszawa, Poland
[3] Department of Medicinal Chemistry, Faculty of Pharmacy, Jagiellonian University Medical College, Medyczna 9, 30-688 Kraków, Poland
[4] Department of Brain Biochemistry, Maj Institute of Pharmacology, Polish Academy of Sciences, Smętna 12, 31-343 Kraków, Poland

Fig. S1A-B; Tab. S1; Tab. S2

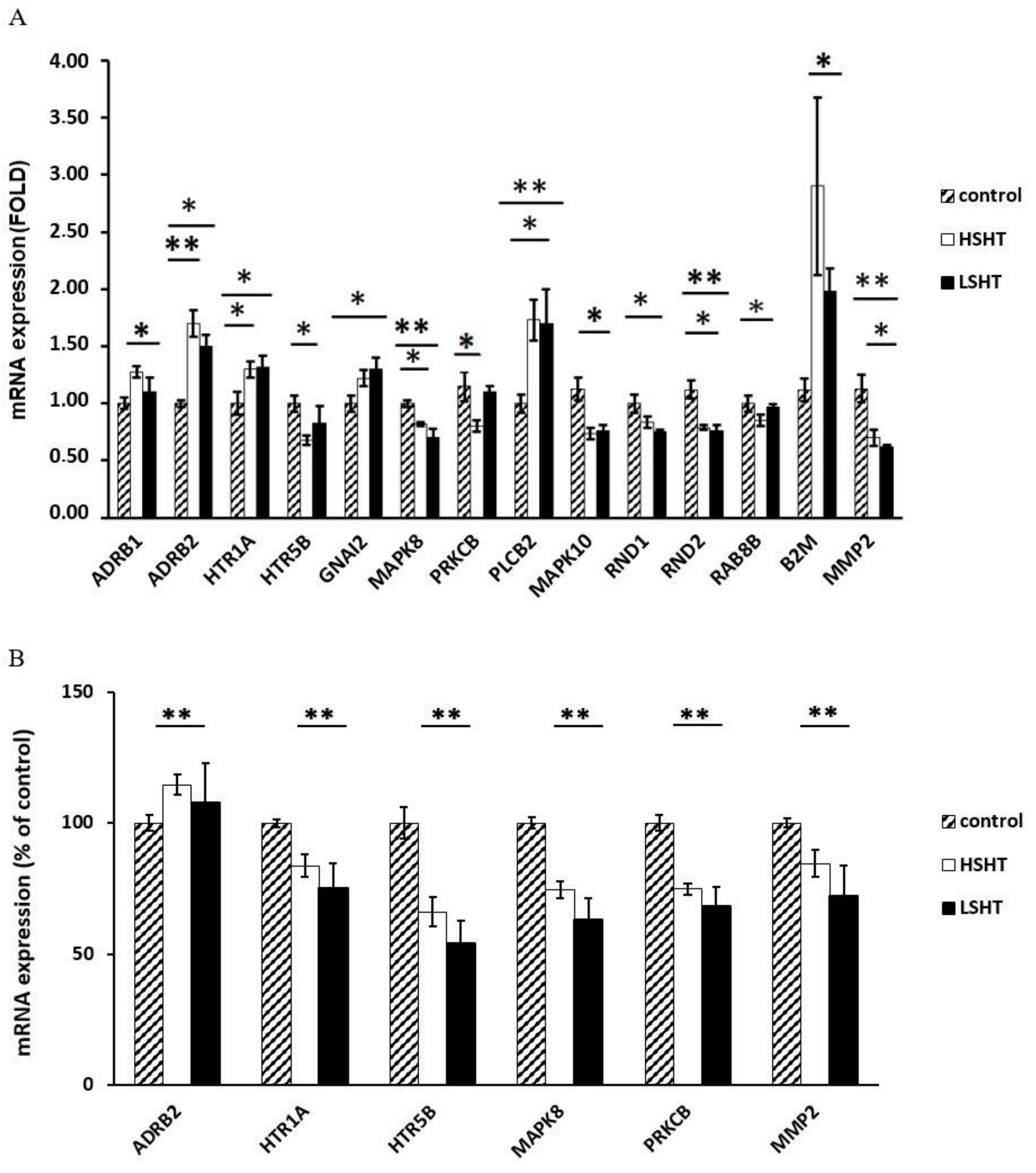

Fig. S1 Gene expression analysis graphs. **A** – Gene expression determined by TaqMan Low Density Array (TLDA). Global normalization was applied (relative to the average expression of all genes). The statistical significance was determined using one-way analysis of variance (one-way ANOVA) and HSD for unequal N (modification of Tukey's test (HSD)): * $P<0.05$, ** $P<0.01$. Control – a group of young rats at 9 weeks of age; HSHT – High Spontaneous Head Twitching rats; LSHT – Low Spontaneous Head Twitching rats; ADRB1 – adrenoceptor β1 gene, ADRB2 – adrenoceptor β2 gene, HTR1A – 5-hydroxytryptamine 1A receptor gene, HTR5B – 5-hydroxytryptamine 5B receptor gene, GNAI2 – guanine nucleotide-binding protein gene, a polypeptide that inhibits the activity of α2, MAPK8 – mitogen-activated protein kinase 8 gene, PRKCB – protein kinase C β gene, PLCB2 – phospholipase C β2 gene, MAPK10 – mitogen-activated protein kinase 10 gene, RND1 – Rho-related GTP-binding protein Rho6 gene, RND2 – Rho-related GTP-binding protein RhoN gene, RAB8B – Ras-related protein Rab-8B gene, B2M – β-2-microglobulin gene, MMP2 – matrix metallopeptidase 2 gene. **B** – Single TaqMan probe assay validation: expression of genes encoding the adrenergic receptor – ADRB2 – β2-adrenergic receptor, serotonin receptors – HTR1A – 5-hydroxytryptamine 1A receptor and HTR5B – 5-hydroxytryptamine 5B receptor, also genes encoding MMP2 – matrix metallopeptidase 2, MAPK8 – mitogen-activated protein kinase 8, PRKCB – protein kinase C β; mRNA expression (% of the control group). The results are presented as the mean ± SEM. The statistical significance was determined using one-way analysis of variance (one-way ANOVA) and Student's t-test for aged rats (HSHT and LSHT) vs young rats (control): ** $P<0.01$. HSHT – High Spontaneous Head Twitching rats (SHT ≥ 7/10 min), LSHT – Low Spontaneous Head Twitching rats (SHT <7/10 min).

Table S1 List of analyzed genes

Assay specific identifier	The full name of the gene	Assay specific identifier	The full name of the gene
Adcy1 Rn02115682_s1	adenylate cyclase 1	Htr5b Rn01408872_m1	5-hydroxytryptamine 5B receptor
Adcy2 Rn00578713_m1	adenylate cyclase 2	Htr6 Rn00577615_m1	5-hydroxytryptamine 6 receptor
Adcy5 Rn00575059_m1	adenylate cyclase 5	Htr7 Rn00576048_m1	5-hydroxytryptamine 7 receptor
Adcy8 Rn00567592_m1	adenylate cyclase 8	Mapk1 (erk2)	mitogen-activated protein kinase1
Adra1a Rn00567876_m1	adrenoceptor α_{1A}	Mapk3 (erk1)	mitogen-activated protein kinase3
Adra1b Rn01471343_m1	adrenoceptor α_{1B}	Mapk7 (erk5)	mitogen-activated protein kinase 7
Adra1d Rn00577931_m1	adrenoceptor α_{1D}	Mapk8 (jnk1) #Rn01218952_m1	mitogen-activated protein kinase 8
Adra2a Rn00562488_s1	adrenoceptor α_{2A}	Mapk9 (jnk2) Rn01464727_m1	mitogen-activated protein kinase 9
Adra2b Rn00593312_s1	adrenoceptor α_{2B}	Mapk10 (jnk3) Rn00689035_m1	mitogen-activated protein kinase 10
Adra2c Rn00593341_s1	adrenoceptor α_{2C}	Mapk11 (p38beta) Rn01407663_g1	mitogen-activated protein kinase 11
Adrb1 Rn00824536_s1	adrenoceptor β_1	Mapk12 (p38gamma) Rn01407421_m1	mitogen-activated protein kinase gene 12
Adrb2 Rn00560650_s1	adrenoceptor β_2	Mapk13 (p38delta) Rn00693138_m1	mitogen-activated protein kinase 13
Adrb3 Rn00565393_m1	adrenoceptor β_3	Mapk14 (p38alpha) Rn00578842_m1	mitogen-activated protein kinase 14
Arf1 Rn01460228_gH	ADP(Adenosine 5′-diphosphate)-ribosylatiofactor 1	Mmp2 Rn01538170_m1	matrix metallopeptidase 2 2
B2m Rn00560865_m1	β-2-microglobulin	Mmp9 Rn00579162_m1	matrix metallopeptidase 2 9
Camk2a Rn01258147_m1	calcium/calmodulin-dependent kinase II α	p11 Rn00821296_g1	calcium binding protein
Camk2b Rn00572627_m1	calcium/calmodulin-dependent kinase II β	Plcb1 Rn01514511_m1	phospholipase C β_1
Camk2d Rn00560913_m1	calcium/calmodulin-dependent kinase II Δ	Plcb2 Rn00585063_m1	phospholipase C β_2
Drd1a Rn03062203_s1	dopamine D_1 receptor	Plcb3 Rn01453968_m1	phospholipase C β_3
Drd2 Rn00561126_m1	dopamine D_2 receptor	Plcb4 Rn00577426_m1	phospholipase C β_4
Drd3 Rn00567568_m1	dopamine D_3 receptor	Prkaca Rn01432300_g1	CAMP (cyclic adenosine-3′, 5′-monophosphate)-activated protein kinase, catalytic subunit α
Drd4 Rn00681263_g1	dopamine D_4 receptor	Prkacb Rn01748540_g1	CAMP-activated protein kinase, β catalytic subunit
Drd5 Rn00562768_s1	dopamine D_5 receptor	Prkar1a Rn00566036_m1	CAMP-dependent protein kinase, α_1 subunit
Gna11 Rn00578959_m1	guanine nucleotide binding protein, α_{11} subunit	Prkar1b Rn01756450_m1	CAMP-dependent protein kinase, the β_1 subunit
Gnai1 Rn00565716_m1	guanine nucleotide binding protein, a polypeptide that inhibits the activity of α_1	Prkar2a Rn00709403_m1	CAMP-dependent protein kinase, α_2 subunit

Gnai2 Rn01447850_m1	guanine nucleotide binding protein, a polypeptide that inhibits the activity of α_2	Prkar2b Rn01473263_m1	CAMP-dependent protein kinase, the β_2 subunit
Gnai3 Rn00565387_m1	guanine nucleotide binding protein, a polypeptide that inhibits the activity of α_{I3}	Prkca Rn01496145_m1	protein kinase C α
Gnao1 Rn00569089_m1	guanine nucleotide binding protein, O_1 subunit	Prkcb Rn00562312_m1	protein kinase C β
Gnaq Rn00578978_m1	guanine nucleotide binding protein, α_Q subunit	Prkcg Rn00440861_m1	protein kinase C γ
Gnas Rn00569454_m1	guanine nucleotide binding protein, stimulating α	Rab8a Rn00588873_m1	oncogene 8A, a member of the RAS family of proteins that control cell growth and death
Gnb1 Rn00515007_m1	guanine nucleotide binding protein, β_1 subunit	Rab8b Rn00596360_m1	oncogene 8B, a member of the RAS protein family
Gnb2 Rn00709509_g1	guanine nucleotide binding protein, β_2 subunit	Rab11a Rn00596360_m1	oncogene 11A, a member of the RAS protein family
Gnb3 Rn00516381_m1	guanine nucleotide binding protein, β_3 subunit	Rab11b Rn00676988_m1	oncogene 11B, a member of the RAS protein family
Gnb4 Rn01755632_m1	guanine nucleotide binding protein, β_4 subunit	Rhoa Rn00589172_m1	small protein B from the a protein family
Gnb5 Rn01521266_m1	guanine nucleotide binding protein gene, β_5 subunit	Rhob Rn00574804_s1	small protein B from the G protein family
Gng12 Rn01425123_m1	guanine nucleotide binding protein, γ_{12} subunit	Rhoc Rn01533495_m1	small protein C from the A protein family
Gng2 Rn01537060_mH	guanine nucleotide binding protein, γ_2 subunit	Rhog Rn02769730_g1	small protein G gene from the a protein family
Gng3 Rn01759733_g1	guanine nucleotide binding protein, γ_3 subunit	Rnd1 Rn01761912_m1	RHO6-related GTP binding protein (*Ras homologous*, belonging to the family of small G proteins)
Hprt Rn01527840_m1	hypoxanthine phosphoribosyltransferase	Rnd2 Rn01249829_gH	RHO7 GTP binding protein related to RHO
Htr1a Rn00561409_s1	5-hydroxytryptamine 1A receptor	Rnd3 Rn01449467_m1	RHO8 GTP binding protein related to RHO
Htr1b Rn01637747_s1	5-hydroxytryptamine 1B receptor	sirt1 Rn01428096_m1	sirtuin 1
Htr1d Rn00563397_s1	5-hydroxytryptamine 1D receptor	sirt2 Rn01457502_m1	sirtuin 2
Htr1f Rn00573147_s1	5-hydroxytryptamine 1F receptor	sirt3 Rn01501410_m1	sirtuin 3
Htr2a Rn00568473_m1	5-hydroxytryptamine 2A receptor	sirt4 Rn01481485_m1	sirtuin 4
Htr2b Rn00691836_m1	5-hydroxytryptamine 2B receptor	sirt5 Rn01450559_m1	sirtuin 5
Htr2c Rn00562748_m1	5-hydroxytryptamine 2C receptor	sirt6 Rn01408249_m1	sirtuin 6
Htr4 Rn00563402_m1	5-hydroxytryptamine 4 receptor	sirt7 Rn01471420_m1	sirtuin 7
Htr5a Rn00565746_m1	5-hydroxytryptamine 5A receptor		

Supplementary Table 2. Summary of the one-way ANOVA results of the mRNA expression of all analyzed genes in the rats' hippocampus (by GenEx Pro 5.7 software).

Adra1a-Rn00567876_m1					
Source	SS	df	MS	F	P-Value
#treat	0,01918372	2	0,00959186	0,1969998	0,82303908
Error	0,82772463	17	0,04868968		
Total	0,84690835	19			
Adra1b-Rn01471343_m1					
Source	SS	df	MS	F	P-Value
#treat	0,16170254	2	0,08085127	1,49395062	0,25251936
Error	0,92002475	17	0,0541191		
Total	1,08172729	19			
Adra1d-Rn00577931_m1					
Source	SS	df	MS	F	P-Value
#treat	0,03740286	2	0,01870143	0,80615247	0,4629291
Error	0,39437244	17	0,02319838		
Total	0,4317753	19			
Adra2a-Rn00562488_s1					
Source	SS	df	MS	F	P-Value
#treat	0,12052635	2	0,06026318	0,97777114	0,39633098
Error	1,04776461	17	0,06163321		
Total	1,16829096	19			
Adra2b-Rn00593312_s1					
Source	SS	df	MS	F	P-Value
#treat	5,51781192	2	2,75890596	3,72345677	0,0455944
Error	12,59619871	17	0,74095287		
Total	18,11401063	19			
Adra2c-Rn00593341_s1					
Source	SS	df	MS	F	P-Value
#treat	0,13876895	2	0,06938448	0,94046553	0,40984239
Error	1,25420447	17	0,07377673		
Total	1,39297342	19			
Adrb1-Rn00824536_s1					
Source	SS	df	MS	F	P-Value
#treat	0,27162202	2	0,13581101	4,2012817	0,03291218
Error	0,54954352	17	0,03232609		
Total	0,82116554	19			
Adrb2-Rn00560650_s1					
Source	SS	df	MS	F	P-Value
#treat	1,81549432	2	0,90774716	12,34956085	0,00048723
Error	1,24957494	17	0,07350441		
Total	3,06506926	19			
Adrb3-Rn00565393_m1					
Source	SS	df	MS	F	P-Value
#treat	0,05986023	2	0,02993011	0,30707911	0,7395878
Error	1,65694095	17	0,09746711		
Total	1,71680118	19			
Drd1-Rn03062203_s1					
Source	SS	df	MS	F	P-Value
#treat	0,08975645	2	0,04487823	1,10821303	0,35285686
Error	0,68843248	17	0,04049603		
Total	0,77818893	19			
18S-Hs99999901_s1					
Source	SS	df	MS	F	P-Value
#treat	0,17776649	2	0,08888324	1,59224492	0,2323618
Error	0,94898411	17	0,05582259		

Total	1,1267506	19			
Drd2-Rn00561126_m1					
Source	SS	df	MS	F	P-Value
#treat	0,22067557	2	0,11033778	2,37532635	0,12311227
Error	0,78967774	17	0,04645163		
Total	1,01035331	19			
Drd3-Rn00567568_m1					
Source	SS	df	MS	F	P-Value
#treat	0,78014929	2	0,39007465	0,26763783	0,76834939
Error	24,77702381	17	1,45747199		
Total	25,55717311	19			
Drd4-Rn00681263_g1					
Source	SS	df	MS	F	P-Value
#treat	115,00600169	2	57,50300085	0,64732059	0,53587404
Error	1510,14973185	17	88,83233717		
Total	1625,15573355	19			
Drd5-Rn00562768_s1					
Source	SS	df	MS	F	P-Value
#treat	0,3251743	2	0,16258715	0,643382	0,53783929
Error	4,29601946	17	0,25270703		
Total	4,62119376	19			
Htr1a-Rn00561409_s1					
Source	SS	df	MS	F	P-Value
#treat	0,43951077	2	0,21975538	5,18263226	0,01748311
Error	0,72083863	17	0,04240227		
Total	1,1603494	19			
Htr1b-Rn01637747_s1					
Source	SS	df	MS	F	P-Value
#treat	0,01862092	2	0,00931046	0,18316323	0,83425374
Error	0,86413522	17	0,05083148		
Total	0,88275613	19			
Htr1d-Rn00563397_s1					
Source	SS	df	MS	F	P-Value
#treat	10,94646157	2	5,47323078	0,7269358	0,49781976
Error	127,9960663	17	7,52918037		
Total	138,94252786	19			
Htr1f-Rn00573147_s1					
Source	SS	df	MS	F	P-Value
#treat	0,12400962	2	0,06200481	1,28006657	0,30349775
Error	0,82345856	17	0,04843874		
Total	0,94746818	19			
Htr2a-Rn00568473_m1					
Source	SS	df	MS	F	P-Value
#treat	0,78042399	2	0,390212	2,38581345	0,12210778
Error	2,78043697	17	0,16355512		
Total	3,56086096	19			
Htr2b-Rn00691836_m1					
Source	SS	df	MS	F	P-Value
#treat	154,25419799	2	77,127099	0,35756624	0,704512
Error	3666,90294744	17	215,70017338		
Total	3821,15714543	19			
Htr2c-Rn00562748_m1					
Source	SS	df	MS	F	P-Value
#treat	0,75386953	2	0,37693476	0,17206228	0,8433747
Error	37,24169428	17	2,1906879		
Total	37,9955638	19			
Htr4-Rn00563402_m1					

Source	SS	df	MS	F	P-Value
#treat	0,19417908	2	0,09708954	1,45516167	0,26100581
Error	1,13425349	17	0,06672079		
Total	1,32843257	19			
Htr5a-Rn00565746_m1					
Source	SS	df	MS	F	P-Value
#treat	5,72612884	2	2,86306442	0,86119488	0,4402962
Error	56,51693537	17	3,32452561		
Total	62,24306421	19			
Htr5b-Rn01408872_m1					
Source	SS	df	MS	F	P-Value
#treat	0,38652272	2	0,19326136	4,98438915	0,01979224
Error	0,65914659	17	0,03877333		
Total	1,0456693	19			
Htr6-Rn00577615_m1					
Source	SS	df	MS	F	P-Value
#treat	0,22527846	2	0,11263923	0,50769407	0,61072406
Error	3,77169447	17	0,22186438		
Total	3,99697293	19			
LOC103694905;Htr7-Rn00576048_m1					
Source	SS	df	MS	F	P-Value
#treat	3,05139136	2	1,52569568	0,9964013	0,38977041
Error	26,03050259	17	1,53120603		
Total	29,08189394	19			
Sirt1-Rn01428096_m1					
Source	SS	df	MS	F	P-Value
#treat	1,41556617	2	0,70778308	2,52531684	0,10958136
Error	4,76467436	17	0,28027496		
Total	6,18024053	19			
Sirt2-Rn01457502_m1					
Source	SS	df	MS	F	P-Value
#treat	0,06857908	2	0,03428954	2,7981581	0,0890217
Error	0,20832353	17	0,01225433		
Total	0,27690261	19			
Sirt3-Rn01501410_m1					
Source	SS	df	MS	F	P-Value
#treat	0,0788968	2	0,0394484	2,47129367	0,11425342
Error	0,27136508	17	0,01596265		
Total	0,35026188	19			
Sirt4-Rn01481485_m1					
Source	SS	df	MS	F	P-Value
#treat	0,50044368	2	0,25022184	2,60124227	0,10337188
Error	1,63528454	17	0,09619321		
Total	2,13572822	19			
Sirt5-Rn01450559_m1					
Source	SS	df	MS	F	P-Value
#treat	0,16204627	2	0,08102314	0,53198116	0,59690494
Error	2,58917685	17	0,15230452		
Total	2,75122312	19			
Sirt6-Rn01408249_m1					
Source	SS	df	MS	F	P-Value
#treat	0,03331193	2	0,01665596	0,29304622	0,74968072
Error	0,96623455	17	0,05683733		
Total	0,99954648	19			
Sirt7-Rn01471420_m1					
Source	SS	df	MS	F	P-Value
#treat	0,07354212	2	0,03677106	4,64886545	0,02451944

Error	0,13446464	17	0,00790968		
Total	0,20800676	19			
Gnas-Rn00569454_m1					
Source	SS	df	MS	F	P-Value
#treat	0,00704595	2	0,00352297	0,18911581	0,82940833
Error	0,31668729	17	0,01862866		
Total	0,32373324	19			
Gnai1-Rn00565716_m1					
Source	SS	df	MS	F	P-Value
#treat	0,07321751	2	0,03660876	1,97771143	0,16896478
Error	0,31468132	17	0,01851067		
Total	0,38789883	19			
Gnai2-Rn01447850_m1					
Source	SS	df	MS	F	P-Value
#treat	0,29889056	2	0,14944528	5,2925014	0,01633409
Error	0,48003195	17	0,02823717		
Total	0,77892252	19			
Gnai3-Rn00565387_m1					
Source	SS	df	MS	F	P-Value
#treat	0,01425709	2	0,00712854	0,34200163	0,71512317
Error	0,35434113	17	0,0208436		
Total	0,36859822	19			
Gnao1-Rn00569089_m1					
Source	SS	df	MS	F	P-Value
#treat	0,04664639	2	0,02332319	0,47570172	0,62947628
Error	0,83349351	17	0,04902903		
Total	0,8801399	19			
Gnaq-Rn00578978_m1					
Source	SS	df	MS	F	P-Value
#treat	0,01565976	2	0,00782988	0,13583745	0,87392259
Error	0,97990616	17	0,05764154		
Total	0,99556592	19			
Gna11-Rn00578959_m1					
Source	SS	df	MS	F	P-Value
#treat	0,02411505	2	0,01205752	0,29618287	0,74741145
Error	0,69206532	17	0,04070972		
Total	0,71618037	19			
Gna12-Rn00667474_m1					
Source	SS	df	MS	F	P-Value
#treat	0,22809188	2	0,11404594	2,06794212	0,15708772
Error	0,93754123	17	0,05514948		
Total	1,16563311	19			
Gnb1-Rn00515007_m1					
Source	SS	df	MS	F	P-Value
#treat	4,26263797	2	2,13131899	0,93383786	0,41229627
Error	38,79947951	17	2,28232232		
Total	43,06211749	19			
Gnb2-Rn00709509_g1					
Source	SS	df	MS	F	P-Value
#treat	0,51186366	2	0,25593183	1,67474756	0,21682527
Error	2,59790861	17	0,15281815		
Total	3,10977227	19			
Gnb3-Rn00516381_m1					
Source	SS	df	MS	F	P-Value
#treat	0,61369738	2	0,30684869	1,550554	0,24068324
Error	3,3642348	17	0,19789616		
Total	3,97793219	19			

Gnb4-Rn01755632_m1					
Source	SS	df	MS	F	P-Value
#treat	0,21344307	2	0,10672154	2,61230903	0,10250009
Error	0,69450669	17	0,04085333		
Total	0,90794976	19			
Gnb5-Rn01521266_m1					
Source	SS	df	MS	F	P-Value
#treat	0,03389639	2	0,0169482	0,53627924	0,59449596
Error	0,53725618	17	0,0316033		
Total	0,57115257	19			
Camk2a-Rn01258147_m1					
Source	SS	df	MS	F	P-Value
#treat	0,15537551	2	0,07768776	1,84963151	0,18758553
Error	0,71402971	17	0,04200175		
Total	0,86940522	19			
Gng12-Rn01425123_m1					
Source	SS	df	MS	F	P-Value
#treat	0,55534536	2	0,27767268	2,60529033	0,10305204
Error	1,81186547	17	0,10658032		
Total	2,36721083	19			
Camk2b-Rn00572627_m1					
Source	SS	df	MS	F	P-Value
#treat	0,09633024	2	0,04816512	0,74623457	0,48905664
Error	1,09725151	17	0,06454421		
Total	1,19358175	19			
Gng2-Rn01537060_mH					
Source	SS	df	MS	F	P-Value
#treat	0,04040587	2	0,02020293	0,2677351	0,76827694
Error	1,28279729	17	0,07545866		
Total	1,32320316	19			
Gng3-Rn01759733_g1					
Source	SS	df	MS	F	P-Value
#treat	0,36230584	2	0,18115292	3,56159911	0,05106453
Error	0,86466769	17	0,05086281		
Total	1,22697353	19			
Camk2d-Rn00560913_m1					
Source	SS	df	MS	F	P-Value
#treat	0,35873112	2	0,17936556	2,01200784	0,16433597
Error	1,51550829	17	0,08914755		
Total	1,87423941	19			
S100a10-Rn00821296_g1					
Source	SS	df	MS	F	P-Value
#treat	1,00515898	2	0,50257949	2,67354931	0,0978219
Error	3,19569618	17	0,18798213		
Total	4,20085516	19			
Mapk8-Rn01218952_m1					
Source	SS	df	MS	F	P-Value
#treat	0,27955642	2	0,13977821	12,67925892	0,0004264
Error	0,18741076	17	0,01102416		
Total	0,46696718	19			
Prkca-Rn01496145_m1					
Source	SS	df	MS	F	P-Value
#treat	0,08617448	2	0,04308724	2,40172501	0,12060116
Error	0,30498207	17	0,01794012		
Total	0,39115655	19			
Prkcb-Rn00562312_m1					
Source	SS	df	MS	F	P-Value

#treat	0,43194171	2	0,21597085	4,16948838	0,03362085
Error	0,88056475	17	0,05179793		
Total	1,31250646	19			
Prkcg-Rn00440861_m1					
Source	SS	df	MS	F	P-Value
#treat	0,05791919	2	0,02895959	0,7322674	0,49538139
Error	0,67231329	17	0,03954784		
Total	0,73023248	19			
Plcb1-Rn01514511_m1					
Source	SS	df	MS	F	P-Value
#treat	0,26224924	2	0,13112462	1,07493092	0,36341918
Error	2,07373187	17	0,12198423		
Total	2,3359811	19			
Plcb2-Rn00585063_m1					
Source	SS	df	MS	F	P-Value
#treat	2,30383232	2	1,15191616	6,3546368	0,00869428
Error	3,08161982	17	0,18127175		
Total	5,38545214	19			
Plcb3-Rn01453968_m1					
Source	SS	df	MS	F	P-Value
#treat	0,25579677	2	0,12789838	1,41470259	0,27019885
Error	1,53691138	17	0,09040655		
Total	1,79270815	19			
Plcb4-Rn00577426_m1					
Source	SS	df	MS	F	P-Value
#treat	0,11035114	2	0,05517557	1,09583483	0,35674457
Error	0,8559544	17	0,05035026		
Total	0,96630554	19			
Adcy1-Rn02115682_s1					
Source	SS	df	MS	F	P-Value
#treat	0,23282725	2	0,11641363	3,72126863	0,04566384
Error	0,53181639	17	0,03128332		
Total	0,76464364	19			
Adcy2-Rn00578713_m1					
Source	SS	df	MS	F	P-Value
#treat	0,06185607	2	0,03092804	1,80674384	0,19432482
Error	0,29100783	17	0,01711811		
Total	0,3528639	19			
Mapk9-Rn01464727_m1					
Source	SS	df	MS	F	P-Value
#treat	0,15296195	2	0,07648098	1,35876754	0,28351011
Error	0,95687935	17	0,05628702		
Total	1,1098413	19			
Mapk10-Rn00689035_m1					
Source	SS	df	MS	F	P-Value
#treat	0,39895297	2	0,19947648	4,78857716	0,02241268
Error	0,70816447	17	0,04165673		
Total	1,10711744	19			
Adcy5-Rn00575059_m1					
Source	SS	df	MS	F	P-Value
#treat	0,37301523	2	0,18650762	0,69393052	0,51321639
Error	4,56908779	17	0,26876987		
Total	4,94210302	19			
Mapk11-Rn01407663_g1					
Source	SS	df	MS	F	P-Value
#treat	0,64299623	2	0,32149812	1,20552479	0,32389122
Error	4,53368361	17	0,26668727		

Total	5,17667984	19			
Mapk12-Rn01407421_m1					
Source	SS	df	MS	F	P-Value
#treat	0,78554801	2	0,392774	0,91245025	0,42032766
Error	7,31783244	17	0,43046073		
Total	8,10338045	19			
Adcy8-Rn00567592_m1					
Source	SS	df	MS	F	P-Value
#treat	0,17518558	2	0,08759279	1,08132231	0,36136372
Error	1,37708938	17	0,08100526		
Total	1,55227497	19			
Mapk13-Rn00693138_m1					
Source	SS	df	MS	F	P-Value
#treat	0,44335919	2	0,2216796	0,09169248	0,9128338
Error	41,09991354	17	2,41764197		
Total	41,54327274	19			
Mapk14-Rn00578842_m1					
Source	SS	df	MS	F	P-Value
#treat	0,40019508	2	0,20009754	2,68068511	0,09729249
Error	1,26895106	17	0,07464418		
Total	1,66914614	19			
Prkaca-Rn01432300_g1					
Source	SS	df	MS	F	P-Value
#treat	0,09071865	2	0,04535933	0,62091831	0,5492032
Error	1,24188405	17	0,073052		
Total	1,3326027	19			
Prkacb-Rn01748540_g1					
Source	SS	df	MS	F	P-Value
#treat	0,06518799	2	0,032594	1,49830314	0,25158649
Error	0,36981698	17	0,02175394		
Total	0,43500498	19			
Prkar1a-Rn00566036_m1					
Source	SS	df	MS	F	P-Value
#treat	0,43996148	2	0,21998074	1,45364017	0,26134513
Error	2,57262608	17	0,15133095		
Total	3,01258756	19			
Prkar1b-Rn01756450_m1					
Source	SS	df	MS	F	P-Value
#treat	0,55442273	2	0,27721136	0,88495688	0,4309099
Error	5,32522351	17	0,31324844		
Total	5,87964624	19			
Prkar2a-Rn00709403_m1					
Source	SS	df	MS	F	P-Value
#treat	0,01776257	2	0,00888128	0,6558193	0,5316607
Error	0,23021866	17	0,01354227		
Total	0,24798123	19			
Prkar2b-Rn01473263_m1					
Source	SS	df	MS	F	P-Value
#treat	0,14067679	2	0,07033839	2,30216789	0,13038212
Error	0,51940292	17	0,03055311		
Total	0,6600797	19			
Rhoa-Rn00589172_m1					
Source	SS	df	MS	F	P-Value
#treat	0,10979453	2	0,05489727	2,66727878	0,09828977
Error	0,34988975	17	0,02058175		
Total	0,45968428	19			
Rhob-Rn00574804_s1					

Source	SS	df	MS	F	P-Value
#treat	0,02948653	2	0,01474326	0,73298799	0,49505285
Error	0,34193671	17	0,02011392		
Total	0,37142324	19			
Rhoc-Rn01533495_m1					
Source	SS	df	MS	F	P-Value
#treat	0,0024728	2	0,0012364	0,01149375	0,98857972
Error	1,82871726	17	0,1075716		
Total	1,83119007	19			
Rnd1-Rn01761912_m1					
Source	SS	df	MS	F	P-Value
#treat	0,22662845	2	0,11331423	6,07080864	0,01024353
Error	0,31731223	17	0,01866543		
Total	0,54394069	19			
Rnd2-Rn01249829_gH					
Source	SS	df	MS	F	P-Value
#treat	0,28172638	2	0,14086319	6,44238116	0,00826975
Error	0,37170638	17	0,02186508		
Total	0,65343276	19			
Rnd3-Rn01449467_m1					
Source	SS	df	MS	F	P-Value
#treat	0,00875497	2	0,00437749	0,08012193	0,92335023
Error	0,92880021	17	0,05463531		
Total	0,93755519	19			
Arf1-Rn01460228_gH					
Source	SS	df	MS	F	P-Value
#treat	0,22434421	2	0,11217211	1,46254039	0,2593672
Error	1,30384487	17	0,07669676		
Total	1,52818908	19			
Rab8a-Rn00588873_m1					
Source	SS	df	MS	F	P-Value
#treat	0,0661451	2	0,03307255	1,31643143	0,29407286
Error	0,42708898	17	0,02512288		
Total	0,49323409	19			
Rab8b-Rn00596360_m1					
Source	SS	df	MS	F	P-Value
#treat	0,10809446	2	0,05404723	4,20908339	0,03274085
Error	0,2182905	17	0,01284062		
Total	0,32638497	19			
Rab11a-Rn00579853_m1					
Source	SS	df	MS	F	P-Value
#treat	0,02602157	2	0,01301078	0,3559516	0,70560456
Error	0,62138599	17	0,03655212		
Total	0,64740756	19			
Rab11b-Rn00676988_m1					
Source	SS	df	MS	F	P-Value
#treat	0,02554517	2	0,01277259	0,33955779	0,71680542
Error	0,63946101	17	0,03761535		
Total	0,66500618	19			
Rhog-Rn02769730_g1					
Source	SS	df	MS	F	P-Value
#treat	0,11115083	2	0,05557541	1,2675851	0,30681009
Error	0,74534012	17	0,04384354		
Total	0,85649095	19			
B2m-Rn00560865_m1					
Source	SS	df	MS	F	P-Value
#treat	15,82588373	2	7,91294187	4,14114023	0,03426713

Error	32,4838098	17	1,91081234		
Total	48,30969353	19			
Mmp2-Rn01538170_m1					
Source	SS	df	MS	F	P-Value
#treat	0,65196192	2	0,32598096	6,6368717	0,0074089
Error	0,83498319	17	0,04911666		
Total	1,48694511	19			
Mmp9-Rn00579162_m1					
Source	SS	df	MS	F	P-Value
#treat	63,75191167	2	31,87595583	1,47876513	0,25580443
Error	366,4484908	17	21,55579358		
Total	430,20040247	19			
Mapk3-Rn00820922_g1					
Source	SS	df	MS	F	P-Value
#treat	0,17291846	2	0,08645923	0,39023152	0,68280983
Error	3,76649968	17	0,2215588		
Total	3,93941814	19			
Mapk1-Rn00671828_m1					
Source	SS	df	MS	F	P-Value
#treat	0,02315234	2	0,01157617	0,14445441	0,86654549
Error	1,36233194	17	0,08013717		
Total	1,38548427	19			
LOC100912585;Mapk7-Rn01460582_g1					
Source	SS	df	MS	F	P-Value
#treat	0,45139865	2	0,22569932	2,05551766	0,15866634
Error	1,86662882	17	0,1098017		
Total	2,31802746	19			
